# mspecLINE: bridging knowledge of human disease with the proteome

**DOI:** 10.1186/1755-8794-3-7

**Published:** 2010-03-10

**Authors:** Jeremy Handcock, Eric W Deutsch, John Boyle

**Affiliations:** 1 Institute for Systems Biology, 1441 N 34th St, Seattle, WA 98103, USA

## Abstract

**Background:**

Public proteomics databases such as PeptideAtlas contain peptides and proteins identified in mass spectrometry experiments. However, these databases lack information about human disease for researchers studying disease-related proteins. We have developed mspecLINE, a tool that combines knowledge about human disease in MEDLINE with empirical data about the detectable human proteome in PeptideAtlas. mspecLINE associates diseases with proteins by calculating the semantic distance between annotated terms from a controlled biomedical vocabulary. We used an established semantic distance measure that is based on the co-occurrence of disease and protein terms in the MEDLINE bibliographic database.

**Results:**

The mspecLINE web application allows researchers to explore relationships between human diseases and parts of the proteome that are detectable using a mass spectrometer. Given a disease, the tool will display proteins and peptides from PeptideAtlas that may be associated with the disease. It will also display relevant literature from MEDLINE. Furthermore, mspecLINE allows researchers to select proteotypic peptides for specific protein targets in a mass spectrometry assay.

**Conclusions:**

Although mspecLINE applies an information retrieval technique to the MEDLINE database, it is distinct from previous MEDLINE query tools in that it combines the knowledge expressed in scientific literature with empirical proteomics data. The tool provides valuable information about candidate protein targets to researchers studying human disease and is freely available on a public web server.

## Background

Public databases of mass spectrometry experiments contain an enormous amount of data about the human proteome. Repositories such as PeptideAtlas [1], PRIDE [2], and Peptidome [3] contain peptides and proteins identified in experiments as well as the empirical evidence to support such identifications. These databases are essential resources for planning mass spectrometry assays, especially in a targeted proteomics workflow where researchers must identify proteotypic peptides for a target protein based on the results of previous experiments [4]. Proteotypic peptides are those peptides that most strongly indicate the presence of a target protein in a sample. They are known to be observable in a mass spectrometer and they map to a unique location in the human genome [5].

Mass spectrometry has significant potential in studying proteins that are involved in human disease. For example, researchers have identified virulence factors of *Streptococcus pyogenes *bacteria [6], candidate biomarkers for erosive rheumatoid arthritis [7], and candidate plasma biomarkers for ovarian cancer [8] using mass spectrometry. Targeted mass spectrometry approaches (*e.g.*, multiple reaction monitoring) can now detect and quantify proteins of very low abundance relative to traditional shotgun approaches (*e.g.*, tandem mass spectrometry) [9], allowing researchers to identify disease-related protein targets with high sensitivity.

Unfortunately, mass spectrometry experiment databases do not currently support searching for disease-related proteins and peptides. Although further integration with genomic databases as in PeptideAtlas [10] could allow researchers to search for proteins as products of a disease-related gene, this would require knowledge of a specific gene that is implicated in a disease. Ideally, proteomics databases should be integrated with knowledge bases of human disease such that researchers may search for candidate protein targets for any disease of interest. We have therefore developed mspecLINE, a tool to assist researchers in exploring the detectable human proteome for disease-related proteins. Using terms from a controlled biomedical vocabulary, mspecLINE associates diseases with sets of proteins by finding the semantic distances between them in previously published scientific literature. mspecLINE allows researchers to enter a disease and view all proteins associated with that disease in the MEDLINE bibliographic database. It also aids researchers in developing targeted assays for disease-related proteins by displaying proteins and peptides from PeptideAtlas that are detectable using the current generation of mass spectrometry instruments.

By combining knowledge about disease with empirical data about the proteome, mspecLINE offers a unique new interface to assist proteomics researchers who are studying proteins that are involved in human disease. We provide an overview of the data sources used by mspecLINE and discuss relevant work on biomedical information retrieval below.

### Data sources overview

mspecLINE incorporates data from a number of sources including the Medical Subject Headings vocabulary, MEDLINE, and PeptideAtlas.

mspecLINE uses the Medical Subject Headings (MeSH) vocabulary to identify semantic relationships between diseases and proteins. MeSH is a controlled vocabulary of biomedical descriptors arranged in a hierarchical structure that can be modeled as a set of trees [11]. MeSH descriptors are assigned to nodes in a particular tree by their subject category. For example, the disease descriptor 'Prostatic Neoplasms' is assigned to a node in the 'Diseases' tree while the enzyme descriptor 'Acid Phosphatase' is assigned to a node in the 'Chemicals & Drugs' tree.

Each reference in the MEDLINE bibliographic database [12] is manually curated and annotated with relevant descriptors from the MeSH vocabulary. For example, an article on Parkinson Disease might be annotated with the MeSH descriptors 'Parkinson Disease' and 'alpha-Synuclein'. mspecLINE analyzes these annotations to associate diseases with proteins. MEDLINE represents an enormous collection of biomedical knowledge about human disease and it currently contains references to more than 16 million articles.

Additionally, mspecLINE uses data from PeptideAtlas, a publicly accessible repository that currently contains empirical data pertaining to more than 130,000 human peptides identified in mass spectrometry experiments. The database also maps peptides to their parent proteins and to their locations in the human genome [10]. Proteins in PeptideAtlas are uniquely identified by accession numbers that can be easily mapped to identifiers used in other protein databases. PeptideAtlas contains high quality data and represents the state of the art in empirical proteomics databases.

### Information retrieval techniques

Investigators have applied numerous information retrieval techniques to extract relationships from biomedical knowledge bases. Many of these techniques involve semantic analysis using Latent Semantic Indexing (LSI) [13]. For example, Homayouni *et al. *[14] successfully used LSI to cluster genes by extracting conceptual relationships from MEDLINE abstracts. Furthermore, Khatri *et al. *used LSI to identify novel gene function annotations by analyzing previous annotations in the human genome [15].

Other investigators have applied co-occurrence analysis techniques to extract relationships from biomedical literature. The Associative Concept Space method, for instance, extracts associations between concepts in literature using co-occurrence data as input [16]. Additionally, Stapley *et al. *developed a method for extracting gene-function relationships from MEDLINE using co-occurrence data [17]. Others have extracted gene and protein synonyms [18] as well as gene clusters [19] from MEDLINE using co-occurrence analysis.

mspecLINE associates diseases with proteins by calculating the semantic distance between MeSH annotations in MEDLINE using Normalized Google Distance (NGD). NGD is grounded in the well-established theory of Kolmogorov complexity [20] and the Normalized Information Distance (NID) between two objects [21]. NID is a general purpose distance measure that is intimately related to coding and compression and it has been successful in a number of contexts including genomics-based phylogeny [22], music clustering [23], and a variety of other applications [24-26]. NGD can be applied to measure the semantic distance between objects in any set of documents. Cilibrasi and Vitányi showed that NGD is effective in distinguishing concepts such as colors and numbers, names of paintings by 17th-century Dutch masters, books by English novelists, and in performing automatic English-Spanish translation. Furthermore, they demonstrated that NGD can be used in a supervised learning setting to accurately classify words into categories in the WordNet database. NGD is similar to LSI in that it can be used to calculate semantic distance between objects, however it is much more computationally feasible than LSI when working with very large document sets such as MEDLINE and it does not require any parameter tuning [27].

NGD is defined as a value between 0 and 1 that indicates the semantic distance between two terms in a set of documents. It uses the co-occurrence of two terms in a document as evidence of their semantic relatedness. If two terms occur frequently in the same documents, those terms are said to be highly related and have a low NGD value. Likewise, if two terms rarely occur in the same documents, those terms are said to be unrelated and have a high NGD value. We apply the NGD measure to the MEDLINE database, so we shall refer to it as Normalized MEDLINE Distance (NMD) throughout this paper. We describe our calculation of NMD in detail in the following section.

## Implementation

mspecLINE builds sets of MeSH disease descriptors and MeSH protein descriptors, then calculates the semantic distances between each pair of descriptors using NMD. We describe our method and provide an overview of the tool's architecture below.

### Building MeSH descriptor sets

First, we used the 2009 MeSH distribution from the National Library of Medicine (NLM) to find all descriptors in the 'Diseases' tree. In total, our set of diseases *D *consisted of 4,323 MeSH descriptors.

We then extracted a list of all unique protein accessions in PeptideAtlas. We used BioThesaurus [28] to find Unified Medical Language System (UMLS) [29] concept annotations for each protein. UMLS is a biomedical vocabulary that is distinct from MeSH. Proteins in BioThesaurus are annotated with UMLS concepts by matching the protein name in public protein databases to a UMLS concept name. Although BioThesaurus provides UMLS concept annotations derived from partial matches to the protein name, we only used annotations that were derived from an exact match. The MeSH distribution from NLM provides a mapping from UMLS concepts to MeSH concepts and using this UMLS-MeSH concept map, we annotated each PeptideAtlas protein with MeSH descriptors that are equivalent to the UMLS concepts in BioThesaurus. We filtered out any MeSH descriptors not in the 'Chemicals & Drugs' tree as we are specifically concerned with those relevant to proteins. Finally, we constructed a set *P *containing 2,585 MeSH protein descriptors to represent all unique annotations for proteins in PeptideAtlas.

### Associating diseases with proteins

To associate the diseases described in MeSH with the proteins in PeptideAtlas, we calculated pairwise semantic distances between each MeSH disease descriptor in *D *and each MeSH protein descriptor in *P *by analyzing article annotations in MEDLINE. We ran our calculations using a local copy of the 2008 MEDLINE database on lease from NLM. For each MeSH disease descriptor *d *∈ *D *and each MeSH protein descriptor *p *∈ *P*, we calculated *NMD*(*d, p*) as:

We define *f *(*d, p*) as the number of articles in MEDLINE that are annotated with both *d *and *p*, and *M *as the total number of articles in MEDLINE. Further, we define *g*_*max*_(*d, p*) and *g*_*min*_(*d, p*) as:

We define *f *(*d*) as the number of articles in MEDLINE that are annotated with *d*, and *f *(*p*) as the number of articles that are annotated with *p*.

**Figure 1:**
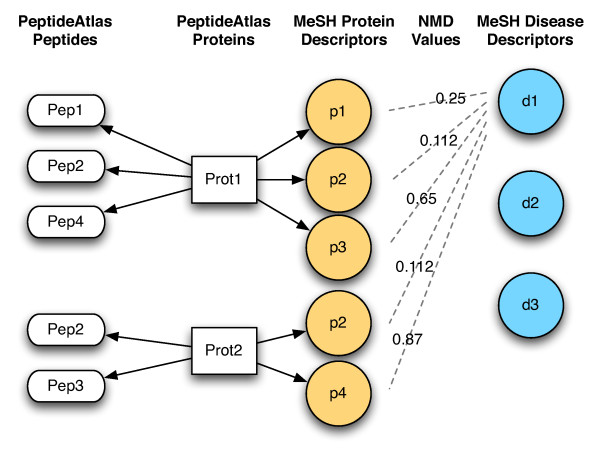
**The associations between diseases, proteins, and peptides in mspecLINE**. First, we constructed sets of all MeSH disease descriptors and all unique MeSH protein descriptors for proteins in PeptideAtlas. Next, we associated diseases with proteins by calculating pairwise NMD values between disease and protein descriptors. Last, we incorporated protein-peptide mappings from PeptideAtlas to construct lists of possible disease-related peptides that are observable in a mass spectrometer.

As shown in Figure 1, each pairwise NMD value associates a MeSH disease descriptor with a MeSH protein descriptor. Together, these associations allow us to find proteins and peptides from PeptideAtlas that may be related to a specific disease.

### Architecture

**Figure 2:**
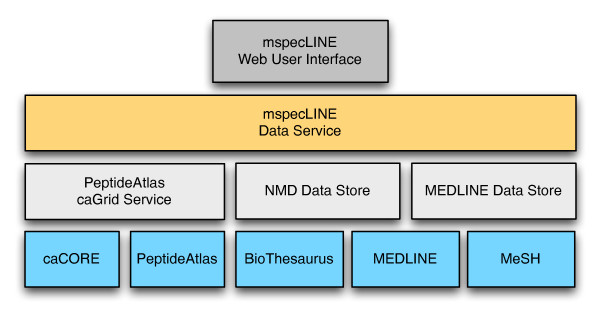
**The high-level architecture of mspecLINE**. Using custom Ruby scripts, we extracted data from BioThesaurus, MEDLINE, and MeSH to construct the NMD and MEDLINE Data Stores in a MySQL database. The mspecLINE Data Service collates protein and peptide information from the PeptideAtlas caGrid Service along with data from the Data Stores. We implemented the Data Service in Java and it presents data to the Javascript-based mspecLINE Web User Interface via a web service. The web service could be used by other applications to access mspecLINE data.

Our software consists of four main components: the NMD Data Store, the MEDLINE Data Store, the mspecLINE Data Service, and the mspecLINE web user interface (UI). Figure 2 shows a high-level view of mspecLINE and its data inputs.

The NMD Data Store has a denormalized schema that encodes the associations depicted in Figure 1. It stores MeSH descriptor pairs along with their NMD values, PeptideAtlas protein identifiers, and PeptideAtlas peptide identifiers. The MEDLINE Data Store contains MeSH descriptor pairs along with PubMed identifiers for articles that are annotated with both descriptors in a pair. The mspecLINE Data Service aggregates data from downstream sources and provides it to the UI via a web service. The Data Service obtains additional protein and peptide information from the PeptideAtlas caGrid service.

### PeptideAtlas caGrid Service

We developed a web service on top of PeptideAtlas that operates on the Cancer Biomedical Informatics Grid with caGrid infrastructure [30]. The PeptideAtlas caGrid Service enables clients like mspecLINE by providing them with detailed information about proteins and peptides in PeptideAtlas. The service has a well-defined query interface that is integrated with the Cancer Common Ontologic Representation Environment (caCORE) [31]. Query-able objects in the PeptideAtlas caGrid Service are annotated with metadata and terms from the caCORE controlled vocabulary, which allows semantic interoperability with other caGrid services and provides clients such as mspecLINE with detailed semantic information about objects in the service interface.

## Results

**Figure 3:**
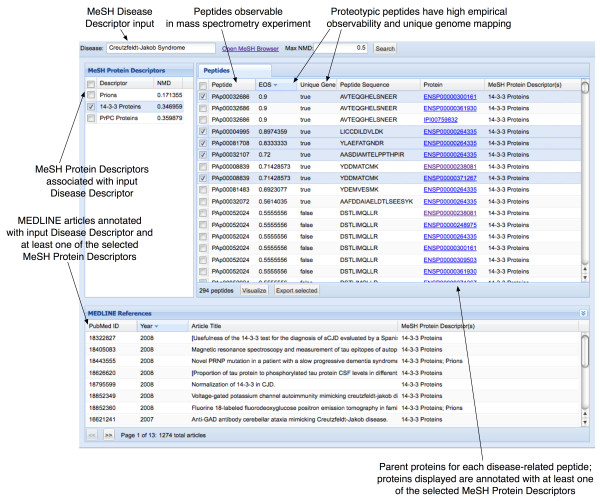
**Screen capture of the mspecLINE web user interface showing Creutzfeldt-Jakob Syndrome as an example disease**. Researchers may review possible disease-related proteins and peptides observable in mass spectrometry experiments, review relevant literature from MEDLINE, and export selected peptides for later use.

Researchers interact with mspecLINE by entering a disease in the UI and viewing associated proteins, peptides, and MEDLINE literature. We provide a walkthrough below using Creuztfeldt-Jakob Syndrome as an example disease. Figure 3 shows a screen capture of the mspecLINE UI and we will refer to it throughout this example.

### Finding disease-related proteins

First, the researcher enters a proper MeSH descriptor name for a disease. He may click on a link to open the NLM MeSH browser and find the proper name. In our example, the MeSH disease name is 'Creutzfeldt-Jakob Syndrome' and mspecLINE finds all matching entries in the NMD Data Store. The UI displays MeSH protein descriptors that are semantically related to Creutzfeldt-Jakob Syndrome in the left panel along with their NMD values: Prions (0.17), 14-3-3 Proteins (0.35), and PrPC Proteins (0.36). The researcher may select or de-select MeSH protein descriptors in the left panel and the other views will filter and update accordingly. It is important to note that mspecLINE only retrieves semantically related protein descriptors below a threshold NMD value. In our example, we have chosen an arbitrary NMD threshold of 0.5, although the UI allows researchers to adjust the threshold.

mspecLINE displays all proteins annotated with one or more of the MeSH protein descriptors in the center panel. The center panel also lists peptides from PeptideAtlas for each protein, the amino acid sequence for each peptide, and the annotated MeSH descriptors for each protein. In our example, mspecLINE found a total of 305 peptides from PeptideAtlas for proteins that may be related to Creutzfeldt-Jakob Syndrome.

The center panel allows the researcher to sort and group peptides by different fields. For example, she may want to find all peptides detectable for a specific target protein related to Creutzfeldt-Jakob Syndrome, therefore she groups peptides by the 'Protein' field.

### Selecting proteotypic peptides

As previously discussed, selecting proteotypic peptides for a protein is an essential step in developing a targeted mass spectrometry assay. mspecLINE displays the PeptideAtlas Empirical Observability Score (EOS) for each peptide in the center panel, which indicates the empirical likelihood that a researcher would observe a specific peptide if its parent protein is detected in the sample [4]. mspecLINE also indicates whether each peptide has a unique mapping in the human genome. The researcher can use this information to select proteotypic peptides for a specific protein target. For example, if he is interested in targeting [IPI:IPI00759832], a protein annotated with the MeSH descriptor '14-3-3 Proteins', he should select peptides with high EOS scores and unique genome mappings such as [PeptideAltas:PAp00032686]. The researcher may export a list of selected peptides from mspecLINE as a tab-separated values file.

### Viewing protein and peptide details

The center panel in the mspecLINE UI displays only minimal information about disease-related proteins and peptides. The researcher may click in the table to open a protein details tab that contains additional information about a protein and its peptides from PeptideAtlas. The information in the tab includes the name of the gene that encodes the protein and a detailed description of the protein. The tab also provides additional empirical information about the protein's peptides including the isoelectric point, molecular mass, and hydrophobicity of each peptide.

### Browsing MEDLINE references

mspecLINE displays all MEDLINE references that contribute to the association of a disease with a set of proteins in the lower panel. The UI queries the MEDLINE Data Store to find all article references that are annotated with both the input MeSH disease descriptor and one or more of the associated MeSH protein descriptors. In our example, mspecLINE found 118 articles in the MEDLINE database that are annotated with the MeSH descriptors 'Creutzfeldt-Jakob Syndrome' and '14-3-3 Proteins'. The researcher can use the MEDLINE references to explore literature that discusses disease-related proteins. She may also click on a specific reference and open the PubMed article entry in a separate browser window. In our example, the MEDLINE references include numerous articles discussing the 14-3-3 brain protein as a clinical marker for Creutzfeldt-Jakob Syndrome in cerebrospinal fluid.

### Visualizing associations

**Figure 4:**
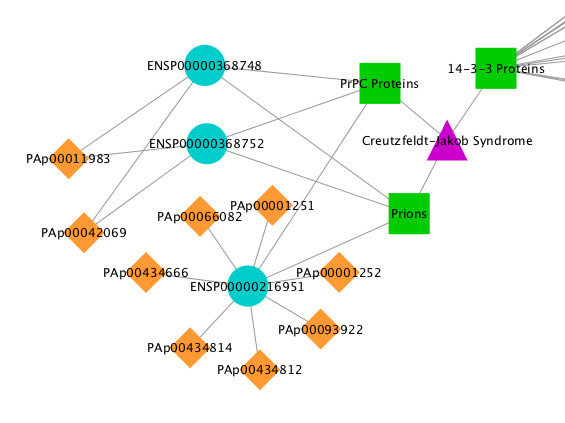
**A portion of the mspecLINE network visualization for Creutzfeldt-Jakob Syndrome**. The visualization allows researchers to see relationships between disease-related proteins and peptides from PeptideAtlas.

In addition to the tabular view in the center panel of the UI, mspecLINE provides a visualization of associations between a disease, MeSH protein descriptors, as well as proteins and peptides from PeptideAtlas. The researcher can click the 'Visualize' button to visualize these associations in a network using Cytoscape [32]. The visualization allows researchers to easily identify relationships between disease-related proteins and peptides from PeptideAtlas. For example, researchers can distinguish peptides that are unique to a specific disease-related protein versus those that may identify multiple disease-related proteins in a mass spectrometry experiment. Figure 4 shows part of the mspecLINE visualization for Creutzfeldt-Jakob Syndrome.

## Discussion

Investigators have developed numerous tools for querying MEDLINE. For example, GoPubMed allows researchers to query and browse MEDLINE using terms from the Gene Ontology [33]. Other tools such as FACTA [34] show semantic relationships in a MEDLINE query result set. Although mspecLINE is similar to these tools in that it applies an information retrieval technique to the MEDLINE database, it is not a generic MEDLINE query tool.

mspecLINE is unique in that it combines knowledge about human disease from MEDLINE with empirical data about the detectable human proteome from PeptideAtlas. It is intended for researchers who study specific diseases and wish to explore candidate proteins for targeting in mass spectrometry experiments. For example, researchers seeking proteins that may be markers for disease could use mspecLINE to identify candidate protein targets and proteotypic peptides for those targets. They could subsequently use analytical tools such as Corra [35] to identify differentially expressed targets among diseased versus non-diseased sample groups. Researchers could also use mspecLINE to browse recent biomedical literature regarding disease-related proteins.

We have identified a number of future enhancements for mspecLINE. First, we are exploring procedures to empirically validate our method for associating diseases with proteins. Second, we are investigating techniques to automatically determine an appropriate threshold for Normalized MEDLINE Distance when a researcher enters a MeSH disease descriptor. Last, we are researching data management systems that would allow us to more effectively store and process the data that we use in our method. Specifically, we are looking at integrating mspecLINE with the Addama infrastructure [36] and developing an automated pipeline to update our Data Stores whenever a new release of MEDLINE is available.

## Conclusions

Mass spectrometry is a promising technology to study proteins involved in human disease. We have presented mspecLINE, a new tool that bridges empirical proteomics data with knowledge about human disease. As we have demonstrated using an example disease, mspecLINE allows researchers to explore potential disease-related protein targets based on semantic distances between disease and protein annotations in MEDLINE. The tool also assists researchers in identifying proteotypic peptides for mass spectrometry assays. mspecLINE is open source software and is freely available for use on a public web server.

## Availability and requirements

• **Project name**: mspecLINE

• **Project home page**: http://informatics.systemsbiology.net/informatics/mspecLINE

• **Operating system(s)**: Platform independent

• **Programming languages**: Ruby, Java, Javascript

• **License**: Apache License, Version 2.0

## Competing interests

The authors declare that they have no competing interests.

## Authors' contributions

JH developed the mspecLINE software, the PeptideAtlas caGrid service, performed data collection and analysis, and drafted the manuscript. ED participated in development of the PeptideAtlas caGrid service. JB initiated and guided the investigation, conceived the application of Normalized Google Distance in our method, and participated in designing the user interface. All authors read and approved the final manuscript.

## Pre-publication history

The pre-publication history for this paper can be accessed here:

http://www.biomedcentral.com/1755-8794/3/7/prepub
